# A community-endorsed open-source lexicon for contrast agent–based perfusion MRI: A consensus guidelines report from the ISMRM Open Science Initiative for Perfusion Imaging (OSIPI)

**DOI:** 10.1002/mrm.29840

**Published:** 2023-10-13

**Authors:** Ben R. Dickie, Zaki Ahmed, Jonathan Arvidsson, Laura C. Bell, David L. Buckley, Charlotte Debus, Andrey Fedorov, Ralf Floca, Ingomar Gutmann, Rianne A. van der Heijden, Petra J. van Houdt, Steven Sourbron, Michael J. Thrippleton, Chad Quarles, Ina N. Kompan

**Affiliations:** 1 Division of Informatics, Imaging, and Data Sciences, School of Health Sciences, Faculty of Biology, Medicine and Health, The University of Manchester, Manchester, UK; 2 Geoffrey Jefferson Brain Research Center, Manchester Academic Health Science Center, The University of Manchester, Manchester, UK; 3 Corewell Health William Beaumont University Hospital, Royal Oak, Michigan, USA; 4 Department of Medical Radiation Sciences, Institute of Clinical Sciences, Sahlgrenska Academy, University of Gothenburg, Gothenburg, Sweden; 5 Department of Medical Physics and Biomedical Engineering, Sahlgrenska University Hospital, Gothenburg, Sweden; 6 Clinical Imaging Group, Genentech, Inc., South San Francisco, California, USA; 7 Biomedical Imaging, University of Leeds, Leeds, UK; 8 Karlsruhe Institute of Technology, Karlsruhe, Germany; 9 Brigham and Women’s Hospital, Harvard Medical School, Boston, Massachusetts, USA; 10 National Center for Radiation Research in Oncology, Heidelberg Institute for Radiation Oncology, Heidelberg, Germany; 11 Faculty of Physics, Physics of Functional Materials, University of Vienna, Vienna, Austria; 12 Department of Radiology & Nuclear Medicine, Erasmus MC University Medical Center, Rotterdam, The Netherlands; 13 Department of Radiology, University of Wisconsin-Madison, Madison, Wisconsin, USA; 14 Department of Radiation Oncology, The Netherlands Cancer Institute, Amsterdam, The Netherlands; 15 Department of Infection, Immunity, and Cardiovascular Diseases, University of Sheffield, Sheffield, UK; 16 Edinburgh Imaging and Center for Clinical Brain Sciences, University of Edinburgh, Edinburgh, UK; 17 Department of Cancer Systems Imaging, UT MD Anderson Cancer Center, Houston, Texas, USA; 18 Division of Medical Image Computing, German Cancer Research Center, Heidelberg, Germany

**Keywords:** CAPLEX, DCE-MRI, DSC-MRI, ISMRM OSIPI, perfusion, standardization

## Abstract

This manuscript describes the ISMRM OSIPI (Open Science Initiative for Perfusion Imaging) lexicon for dynamic contrast-enhanced and dynamic susceptibility-contrast MRI. The lexicon was developed by Taskforce 4.2 of OSIPI to provide standardized definitions of commonly used quantities, models, and analysis processes with the aim of reducing reporting variability. The taskforce was established in February 2020 and consists of medical physicists, engineers, clinicians, data and computer scientists, and DICOM (Digital Imaging and Communications in Medicine) standard experts. Members of the taskforce collaborated via a slack channel and quarterly virtual meetings. Members participated by defining lexicon items and reporting formats that were reviewed by at least two other members of the taskforce. Version 1.0.0 of the lexicon was subject to open review from the wider perfusion imaging community between January and March 2022, and endorsed by the Perfusion Study Group of the ISMRM in the summer of 2022. The initial scope of the lexicon was set by the taskforce and defined such that it contained a basic set of quantities, processes, and models to enable users to report an end-to-end analysis pipeline including kinetic model fitting. We also provide guidance on how to easily incorporate lexicon items and definitions into free-text descriptions (e.g., in manuscripts and other documentation) and introduce an XML-based pipeline encoding format to encode analyses using lexicon definitions in standardized and extensible machine-readable code. The lexicon is designed to be open-source and extendable, enabling ongoing expansion of its content. We hope that widespread adoption of lexicon terminology and reporting formats described herein will increase reproducibility within the field.

## 1 ∣ INTRODUCTION

Dynamic contrast-enhanced (DCE) and dynamic-susceptibility contrast (DSC) MRI are regarded as the primary contrast-agent based MRI modalities for imaging tissue perfusion. Perfusion quantities derived from DCE-MRI and DSC-MRI have potential as quantitative imaging biomarkers (QIBs) in applications such as oncology, neurology, cardiology, liver, renal, and musculoskeletal disorders^1^; however, translation of these QIBs to the clinic is hindered by lack of standardization in both acquisition and analysis procedures. Despite the best efforts of the research and clinical communities, there remains substantial interstudy variability in the reported values of DCE/DSC MRI QIBs, with data analysis being a major contributor to this variation.^2,3^

DCE and DSC-MRI data are typically analyzed using a sequence of computational processes which define an image analysis pipeline. The specific steps included within these pipelines, and the software used, can be highly variable from study to study and between research groups. Clinically oriented studies often rely on commercial software. Several studies have found substantial differences and inconsistencies between these tools^4-7^—the details of which are rarely fully documented by the vendor. When commercial solutions are not available, or do not have the desired functionality, in-house software is often developed, which can result in very different implementations of the same basic analysis pipeline, varying in factors such as the coding language used (*Python, MATLAB, C++*), curve-fitting algorithm and tuning parameters, output data type and units, and the overall choice and number of analysis steps.

There have been many efforts to set standards in medical imaging, including initiatives to improve data standards such as the DICOM (Digital Imaging and Communications in Medicine) standard^8^ and BIDS (Brain Imaging Data Structure),^9^ and there are a number of more specific initiatives focused around metrology standards for QIBs^10^ and standardized methods and results reporting for neuroimaging (COBIDAS,^11^ NIDMTerms^12^). Guidance on the acquisition of perfusion MRI for multicenter studies has been established for many years through the RSNA QIBA (Quantitative Imaging Biomarkers Alliance), which have published a number of recommended protocols focused on acquisition of DCE and DSC data.^13,14^ Similar efforts have been made for arterial spin-labeling MRI in brain^15^ and kidney.^16,17^ However, very little guidance has been developed on how to report analysis methods and results, or recommended terminology for perfusion quantities, models, or processes.

To address this, Taskforce 4.2 of the ISMRM Open Science Initiative for Perfusion Imaging (OSIPI) was established to develop a lexicon of standard terminology for DCE/DSC MRI. OSIPI’s overall mission is to promote the sharing of perfusion imaging software, improve the reproducibility of perfusion imaging research, and speed up the translation into tools for discovery science, drug development, and clinical practice. This manuscript describes the main deliverable from the first 2-year cycle (2020–2022) of Task Force 4.2: an open-source DCE/DSC lexicon of standard quantities, models, and processes (the contrast agent-based perfusion lexicon [CAPLEX]).

## 2 ∣ ESTABLISHMENT OF OSIPI TASKFORCE 4.2

Taskforce 4.2 was established in February 2020 by the executive management board of OSIPI. At the time of writing, the taskforce consists of a lead (IK), co-lead (BD), and contributing scientists with backgrounds in medical physics and engineering, image analysis, and the DICOM standard. Participation is open to any individual with relevant expertise, and those interested in contributing are invited to contact the taskforce or OSIPI management. Members of the taskforce collaborated via a slack channel and regular (quarterly) virtual meetings. The activity of the taskforce followed a predefined roadmap that was agreed between the co-leads and the executive management board. Tasks during the first 2-year cycle of OSIPI (2020–2022) were focused primarily on five milestones, culminating in the production of v1.0.2 of the lexicon described herein.

## 3 ∣ CAPLEX STRUCTURE, DEFINITIONS, AND CURRENT SCOPE

### 3.1 ∣ Accessing CAPLEX

The most up-to-date version of the lexicon can be accessed on the CAPLEX webpage at https://osipi.github.io/OSIPI_CAPLEX. The source code driving the webpage is maintained on GITHUB (https://github.com/OSIPI/OSIPI_CAPLEX), which is used for development and deployment of CAPLEX content. The version of the lexicon at the time of writing is v.1.0.2 (10.5281/zenodo.8180201).

### 3.2 ∣ Structure and definitions

CAPLEX is organized into five sections, which group together similar quantities, models, or processes.

Quantities (Section Q; https://osipi.github.io/OSIPI_CAPLEX/quantities/) represent variables that usually act as input or output to an analysis process, such as the longitudinal relaxation rate, $R_{1}$.

Processes (Section P [https://osipi.github.io/OSIPI_CAPLEX/perfusionProcesses/], Section G [https://osipi.github.io/OSIPI_CAPLEX/generalPurposeProcesses/] and Section D [https://osipi.github.io/OSIPI_CAPLEX/derivedProcesses/]) are operators that generate output quantities from given input quantities. They define semantically what is done by each analysis step, in a similar, way to a function or routine name within a programming environment but provide only high-level detail of the implementation. For example, Variable Flip Angle is a process that maps variable-flip-angle signal intensities to the longitudinal relaxation rate and equilibrium magnetization. All required inputs to a process (quantities, models, and other processes) are defined by the lexicon entry. This approach minimizes the risk that the user will fail to report all the relevant information.

Models (Section M; https://osipi.github.io/OSIPI_CAPLEX/perfusionModels/) are mathematical formulas that relate quantities to one another and are typically used to describe the functional behavior of measured data. It is common to fit these models to data using an optimizer, with either free or fixed parameters. Because the choice of parameters to fix or keep free during optimization may vary, we specify only the model formula and quantities involved, leaving the user to specify which are free or fixed using the model parameters (free) and static model parameters (fixed) quantities, respectively. Because models often have multiple possible parameterizations (particularly true for kinetic models), we aim to define models using a harmonized parameterization such that different models can be easily compared, and to improve consistency of reporting.

**FIGURE 1:**
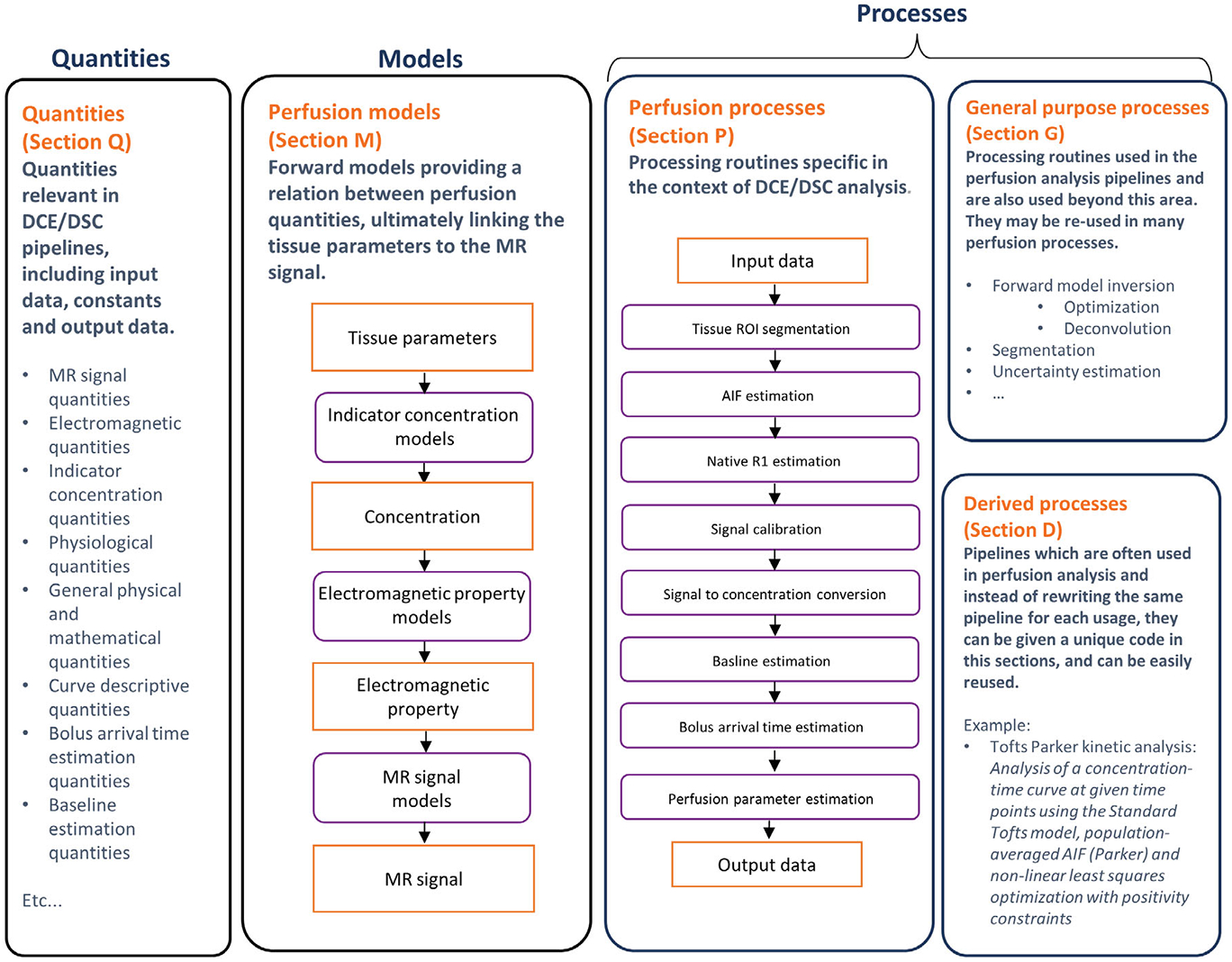
Organization of CAPLEX sections: quantities (Section Q), perfusion models (Section M), perfusion processes (Section P), general purpose processes (Section G), and derived processes (Section D). In the flow charts, quantities are given in orange boxes, and processes and models are shown in purple boxes. The pipeline shown in the perfusion processes box represents a typical pipeline that can be encoded using quantities, processes, and models currently defined within CAPLEX version 1.0.2. It is possible to encode pipelines with fewer or entirely different processes than those shown (e.g., using $R_{2}$ or $R_{2}^{\;\;*}$ estimation and leakage correction when reporting DSC-MRI analyses). AIF: arterial input function; DCE: dynamic contrast enhanced; DSC: dynamic susceptibility contrast.

Each section contains a list of items arranged in thematic groups. An overview of the sections and their content is provided in Figure 1.

Each item is identified by a unique code (e.g., Q.MS1.001.[j]), which is structured as Section.Group.Item.[CompartmentCode], and which is intended to be machine-readable. For instance, Q.MS1.001.[a] is item 001 from group MS1 in section Q. The [a] defines the compartmental origin of the quantity as being from an artery. Each lexicon item has an OSIPI name and notation. The OSIPI name provides an OSIPI-recommended, human-readable name (e.g., indicator concentration), for which the aim is to be as general as possible, avoiding anatomy-specific terminology (such as *cerebral* blood flow). The use of a single standardized name for a given quantity should reduce confusion regarding the meaning of parameters within the community. The notation gives the symbolic or abbreviated form for use in equations or shorthand. If the quantity is known to vary between tissue compartments (e.g., $R_{1}$, which varies between blood and tissue), then it is defined in the lexicon with an *optional* CompartmentCode. When the user wishes to report the quantity of interest, they should assign the appropriate character subscript from Table 1.

Relationships between compartment indices can be expressed in three ways:

1. When referring to a smaller compartment within a larger compartment, comma separation between the two compartment subscripts should be used, with the larger compartment appearing first. For example, arterial plasma is written as “a,p.”
2. When one wants to report a quantity that relates to a combination of compartments, the compartment subscripts should be written together without comma or whitespace separation. For example, the combined blood and extravascular extracellular compartment is written as “be.”
3. When expressing exchange from one compartment to another, an arrow, →, is used to denote the direction of exchange. For example, the water exchange rate from the extravascular extracellular compartment to the intracellular compartment is written as $k_{w,e\to i}$.

To provide some clarity on the meaning of each quantity, model or process, a set of alternative names can be optionally specified alongside a free-text description. Alternative names should be provided particularly for quantities, models, or processes that have historically been referred to by a number of different names in the literature. The description should provide sufficient information to unambiguously define the quantity, model or process, but should not contain unnecessary detail. For processes, the description details all inputs and outputs, where inputs and outputs are stated as a complete, ordered list of quantities, models and (other) processes, which are defined by their unique code (and optionally their OSIPI name for human readability).

All lexicon quantities are assigned an OSIPI unit using the International System of Units (SI). All tissue quantities are normalized to tissue volume (i.e., 100 mL) rather than tissue mass (i.e., 100 g), as these are the natural units for imaging data.

Finally, an original reference can be optionally provided. Examples of lexicon entries for plasma flow and the standard Tofts model can be found in Tables 2 and 3, respectively.

In summary, an item of the lexicon is characterized by the following elements:

1. Code (required): a unique identifier structured as [Section.Group.Item.CompartmentCode]. The CompartmentCode is used only for (certain) quantities if it is required to specify the compartment, subcompartment of origin, or exchange from one compartment to another.
2. OSIPI name (required): a name as recommended by OSIPI.
3. Alternative names (optional): a set of alternative names used in the literature to help put the definition into context.
4. Notation (required): short notation for reporting brevity.
5. Description (required): A description providing sufficient details to precisely define the quantity, model or process.
  For processes, inputs and outputs must be specified as a complete, ordered list of quantities, models and/or other processes and each defined by their code and optionally their OSIPI name.
6. OSIPI unit (required): For quantities, an OSIPI-endorsed unit should be defined.
7. Reference (optional): A reference to original publication supporting the OSIPI-adopted definition of the quantity, process, or model.

### 3.3 ∣ Current scope of CAPLEX

#### 3.3.1 ∣ Quantities

The Quantities section contains quantities commonly used in DCE and DSC-MRI analyses and include MR signal quantities, electromagnetic quantities, indicator concentration quantities, physiological quantities, general physical and mathematical quantities, curve descriptive quantities, bolus arrival time estimation quantities, baseline estimation quantities, descriptive model quantities, leakage correction model quantities, uncertainty and statistical quantities, analytical inversion quantities, optimization quantities, native $R_{1}$ estimation quantities, arterial input function quantities, and segmentation quantities.

#### 3.3.2 ∣ Models

The Models section contains relevant models including the general forward model, signal models, electromagnetic property models, indicator concentration models, descriptive models, heuristic models, and perfusion identity models.

#### 3.3.3 ∣ Processes

Processes are divided into the three sections:

- Perfusion processes (Section P) containing processes specific to DCE/DSC MRI, including native $R_{1}$ estimation methods, bolus arrival time estimation methods, baseline estimation methods, signal scaling factor estimation methods, arterial input function estimation methods, signal to concentration conversion methods, signal to electromagnetic property conversion methods, electromagnetic property to concentration conversion methods, leakage correction methods, and parameter extraction methods.
- General purpose processes (Section G) containing processes that are often used in perfusion analyses but are also used beyond this area, including forward model inversion methods, optimization methods, deconvolution methods, discretization methods, curve descriptive processes, segmentation processes, uncertainty estimation and statistical processes, and averaging methods.
- Derived processes (Section D) containing composite processes made up of processes from Sections P and G. Processes in Sections P and G are primary processing units required to conduct DCE/DSC analysis pipelines. It may be useful to combine multiple primary processes into a single composite process, and then store the result as a new process, which we refer to as a derived process. For analysis pipelines that are repeatedly used (e.g., in multiple publications following the same protocol, multicenter studies, or for standard use of an open-source software), use of derived processes will avoid reporting duplication and minimize reporting variability.

### 3.4 ∣ Editing or adding entries to CAPLEX

CAPLEX is an open-source community resource and is designed to be extendable. As such, we encourage users to actively contribute to CAPLEX as well as provide feedback on existing entries (e.g., including refining descriptions, notation etc.).

Suggested edits or additions to CAPLEX can be made by:

1. Contacting a current member of OSIPI Taskforce 4.2 with your query, who will review the request and submit a change request on your behalf.
2. Logging an issue on the CAPLEX GITHUB repository (https://github.com/OSIPI/OSIPI_CAPLEX/issues).
3. Creating a fork of the GITHUB repository, editing the relevant .md files (found in /docs directory of the main branch), committing the changes, and submitting a pull request. The taskforce will aim to review the requested changes within 10 working days. Once reviewed and approved, the committed changes will be merged with the original CAPLEX repository and automatically deployed to the lexicon webpage.

### 3.5 ∣ Guidelines for reporting DCE/DSC methods and results using CAPLEX

In addition to providing the lexicon itself, we also propose guidance and tools to help users incorporate the lexicon into their manuscripts and other documentation. The simplest way users can conform to the lexicon standard is to ensure that quantities, models, and processes are referred to with their OSIPI name or notation (or both) and to insert a hyperlink to the correct entry on the lexicon webpage. For quantities, units must also be specified. This approach, which we call lexicon-linked free-text format, does not require much additional effort on behalf of the user other than searching the lexicon for the correct term and inserting the relevant hyperlink.

The second approach, which we call the lexicon-linked XML pipeline format, requires a greater investment in time and effort from the user, but allows a much more complete, standardized, and structured description of the analysis performed. The aim here is to provide a means for users to record their analysis pipelines in a precise structured manner that avoids ambiguity. Instead of developing a new encoding format, we have harnessed the widely used eXtensible Markup Language (XML). A key benefit of XML is its extensibility (which conforms to a key design feature of CAPLEX) and the widespread availability of open-source XML parsers, meaning that lexicon-linked XML pipelines can be read by any machine or software. Furthermore, by using XML schema definition files, it is possible to set “encoding rules”. This is important, as it allows the lexicon management team to define the allowable ways in which a user can encode a pipeline (e.g., it would not be possible to use a quantity as the first item within the pipeline). In practical terms, these schema files help the user to write “valid” XML pipelines that conform to CAPLEX standards.

To achieve the aims of OSIPI, we strongly encourage that all manuscripts and documentation that describe DCE/DSC MRI experiments/studies should—as a minimum—use lexicon-linked free-text when reporting both methods and results. Use of the lexicon-linked XML pipeline format in addition is not as essential but could be used when a more precise description of the methods is required. When reporting using either format, users should write the following statement in their manuscripts: “All quantities, models and processes conform to OSIPI CAPLEX (version xxx)”, and cite this article.

### 3.6 ∣ Lexicon-linked free-text format

When reporting methods using the lexicon-linked free-text format, users should report as they normally would, with the following additional requirements:

1. All quantities, models, and processes should be defined using their OSIPI name or notation (or both).
2. All quantities, models, and processes should be hyperlinked to their entry in the lexicon. Details on how to obtain the correct hyperlink are given on the GITHUB README (https://github.com/OSIPI/OSIPI_CAPLEX).
3. All quantities should be defined with their OSIPI unit (e.g., $K^{\text{trans}}$ [min^−1^]).
4. All practicable attempts should be made to describe every executed process, regardless of how trivial they may seem. This includes descriptions of how uncertainties on quantities are calculated.

We recommend that estimated values of quantities and uncertainties on those quantities be reported in the following manner:

OSIPIname∕notation=quantity value±quantity uncertaintyOSIPIunit.

### 3.7 ∣ Use case for reporting DCE/DSC methods using lexicon-linked free-text

Here we provide a use case of the lexicon-linked free-text format applied to the postprocessing section of Sourbron et al.^18^ We have adapted the manuscript text to comply with CAPLEX, including replacing quantity, models, and process names with OSIPI names and hyperlinking these to their entry within the lexicon webpage (https://osipi.github.io/OSIPI_CAPLEX/). In contrast to convention, hyperlinks within lexicon-linked text are given the same color as non-hyperlinked text such that they are not distracting to the reader. Please refer to the article for the original text.

#### 3.7.1 ∣ Excerpt adapted from Sourbron et al. 2009^18^

All quantities, models, and processes conform to OSIPI CAPLEX (v.1.0.2). A map of the baseline signal $S_{\text{BL}}$ (a.u) was calculated as the sample arithmetic mean of the first 15 to 20 dynamics. A binary tissue region-of-interest (ROI) mask was created semi-automatically to segment out background by setting a threshold on the $S_{\text{BL}}$ (a.u) values and disregarded in all subsequent calculations. The area under the relative enhancement (a.u) curves (AUC_0,420 s_ [a.u]) was calculated on the pixel level. A venous voxel was identified as the pixel in the sinus with the maximum AUC_0,420 s_ (a.u). On the lowest axial slice of the AUC_0,420 s_ (a.u) map, two circular ROIs were drawn freehand around the internal carotid arteries. The six pixels within those circles with maximum AUC_0,420 s_ (a.u) were selected automatically, and their signal-time curves were averaged using the arithmetic mean to extract the arterial whole blood signal, $S_{a,b}$ (a.u). These steps were repeated for all data by two independent observers (S.S. and M.I.) to assess the reproducibility of this approach to selecting $S_{a,b}$ (a.u).

The impulse response function I(t)=PA⋅H(t) in Eq. (31) was determined by model-free deconvolution with generalized cross-validation and integrated to produce an arterial partial-volume correction factor PA (unitless). A map of native longitudinal relaxation rate $R_{10}\;(s^{-1})$ was calculated by fitting the saturation-recovery data with variable prepulse delay times (ms) to a monoexponential. The change in longitudinal relaxation rate $\Delta R_{1}\;(s^{-1})$ was calculated for every voxel with Eq. (27), assuming a linear model between signal S (a.u) and $R_{1}\;(s^{-1})$. The native longitudinal relaxation rate in venous whole blood $R_{10,v,b}\;(s^{-1})$ was calculated from the $R_{10}$-value $\left(s^{-1}\right)$ in the venous voxel, and the change in longitudinal relaxation rate in arterial whole blood $\Delta R_{1,a,b}\;(s^{-1})$ was calculated with Eq. (32).

The change in the longitudinal relaxation rate of tissue $\Delta R_{1,t}\;(s^{-1})$ and arterial blood $\Delta R_{1,a,b}\;(s^{-1})$ was fit pixel-by-pixel to the two compartment uptake model, producing maps of $F_{b}$ (mL/min/100 mL), ${\text{MTT}}_{c,p}\;\;(s)$, and E (unitless). To calculate the permeability surface area product PS (mL/min/100 mL) from Eq. (19), the blood plasma flow $F_{p}$ (mL/min/100 mL) was calculated as ($1-{\text{Hct}}_{a}$ (unitless)) $\cdot F_{b}$ (mL/min/mL) with a fixed arterial hematocrit value of ${\text{Hct}}_{a}=0.45$ (unitless). A binary tissue ROI mask covering the lesion was drawn manually on the PS map (mL/min/100 mL) and superposed on the $F_{b}$ map (mL/min/100 mL) to exclude areas of necrosis and larger blood vessels. Contralateral to the lesion, circular white-matter and gray-matter tissue ROI masks were defined on the map of the baseline signal $S_{\text{BL}}$ (a.u), superposed on the $F_{b}$ map (mL/min/100 mL) and shifted if necessary to exclude larger blood vessels.

The analysis was then repeated on the ROI level. The tumor ROI data were fitted both with the two-compartment uptake model and with the two-compartment exchange model, and the Akaike information criterion was used to select which of both models was most appropriate for the ROI data. Model selection was verified by visual inspection of the goodness-of-fit of both models. Model fitting was performed using MPFIT, with defaults for the maximum number of iterations and convergence threshold, without constraints or limits on the parameters, and with analytical formulas for the partial derivatives. The initial values were fixed to $F_{p}=2$ mL/min/100 mL, PS = 0.2 mL/min/100 mL, $v_{p}=10$ mL/min/100 mL, and $v_{e}=20$ mL/min/100 mL.

We include the relevant equations (original and adapted to conform with CAPLEX terminology) as follows, for reference.

**Table:** 

Original equation from Sourbron (2009)	Adapted equation to conform with CAPLEX terminology	Equation No.
$F_{E}=EF_{p}∕(1-E)$	$\text{PS}=EF_{p}∕(1-E)$	19
$\Delta R_{1}=R_{10}\frac{\Delta S}{S_{0}}$	$\Delta R_{1}=R_{10}\frac{\Delta S}{S_{\text{BL}}}$	27
$\frac{\Delta S_{v}}{S_{0,v}}=P_{A}H⊗\frac{\Delta S}{S_{0}}$	$\frac{\Delta S_{v}}{S_{\text{BL},v}}=P_{A}H⊗\frac{\Delta S_{a,b}}{S_{\text{BL},a,b}}$	31
$\Delta R_{1,A}=P_{A}R_{10,B}\frac{\Delta S}{S_{0}}$	$\Delta R_{1,a,b}=P_{A}R_{10,v}\frac{\Delta S_{a,b}}{S_{\text{BL},a,b}}$	32

### 3.8 ∣ Lexicon-linked XML pipeline format

Although the lexicon linked free-text format provides a mechanism to link quantities, models, and processes to standard lexicon definitions using standard free-text formats, it still requires the user to choose the analysis steps to report and in which order to report them. When reporting a DCE/DSC analysis, it is crucial that the steps are accurately recorded, and that sufficient detail is provided. This is often at the discretion of the researcher, and without prompts for which quantities or parameters to report, it is difficult for the user to know what detail is necessary, particularly for those with little experience. To help address these issues, we propose the lexicon-linked XML (LL-XML) pipeline format, which harnesses the extensibility and rule-making properties of XML and XML schema. The LL-XML pipeline format has the following advantages over lexicon-linked free-text:

1. Analysis pipelines are reported with a predefined set of commands and rules defined within an XML schema, leading to a reproducible standardized reporting format;
2. The XML format is machine-readable with existing open-source parsers. This provides the possibility for LL-XML pipeline files to be parsed by computers (e.g., as a .config file to DCE/DSC MRI analysis software) or for machine-learning applications.

Within the proposed LL-XML pipeline format, a single process is encoded as follows:


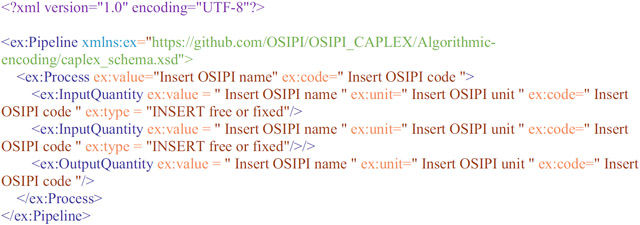


The opening and closing ex:Pipeline commands define the start and end of the pipeline, as well as the namespace (ex) and schema (.xsd) file against which the pipeline is validated. The schema file is hosted on https://github.com/OSIPI/OSIPI_CAPLEX, and a local copy should be stored in the user’s local CAPLEX repository in the same directory as the XML pipeline file. To comply with the schema rules, the pipeline command must be followed by at least one child process. Processes can have the following child elements: another different Process, InputQuantity, OutputQuantity or Model (in any order). Each element can be repeated as often as necessary. Processes must be defined with *value* and *code* attributes, which should be set to the OSIPI name and OSIPI codes, respectively. The XML file will not validate if the OSIPI names and OSIPI code do not match. They can also have optional *instance* attributes, which describe detail of the specific implementation, such as using the Numpy function. InputQuantities and OutputQuantities can have *value, unit, code,* and optional *instance* and *type* attributes. An optional *type* attribute is used to define whether an InputQuantity is “free” or “fixed” during an optimization. Often it is necessary to define assumptions in the form of mathematical relations. We provide a mechanism to report this explicitly with the <SetEqual> command. An example is given in the encoding example given subsequently.

## 4 ∣ USE CASE FOR REPORTING METHODS USING LEXICON-LINKED ALGORITHMIC ENCODING

For completeness, we provide an example of the lexicon-linked XML pipeline format applied to an excerpt (Paragraph 4) of the postprocessing section of Sourbron et al. (2009).^18^


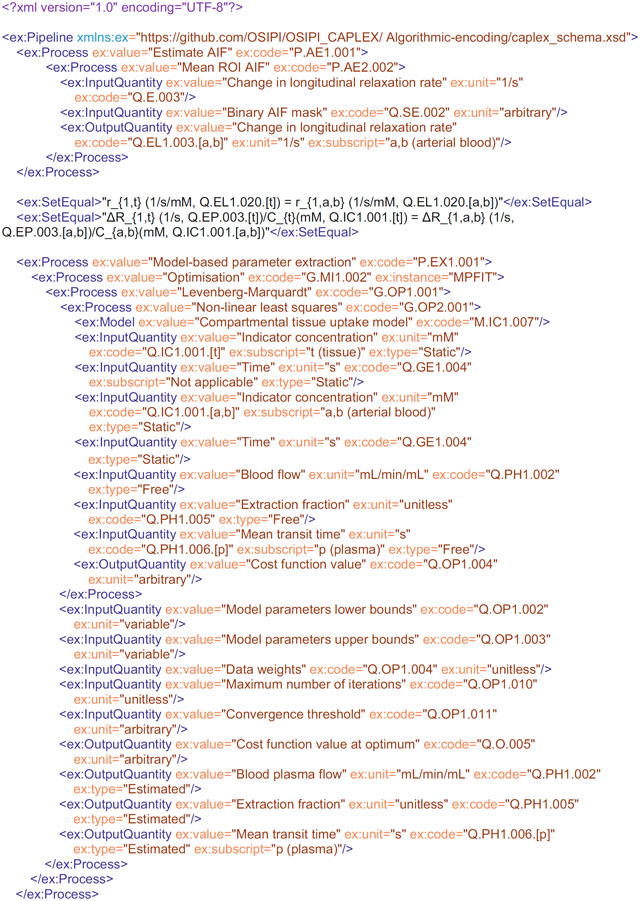



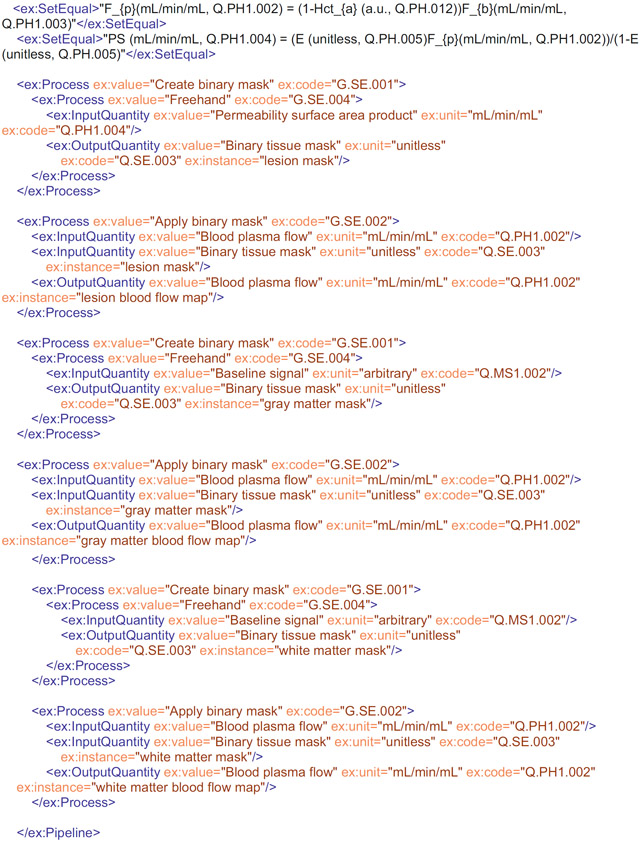


## 5 ∣ DISCUSSION AND OUTLOOK

We have presented a community-endorsed open-source lexicon for DCE/DSC MRI. The aim of the lexicon is to increase reproducibility of contrast-agent based perfusion MRI, specifically to improve standardization by reducing reporting variability. The lexicon includes commonly used quantities, models and analysis processes that have been compiled by perfusion MRI experts within OSIPI Taskforce 4.2. The entries have been subject to scrutiny by the wider perfusion community and endorsed by the perfusion study group of the ISMRM. The CAPLEX definitions of quantities, models, and processes should now be adopted and incorporated into future DCE/DSC manuscripts and documentation to increase reproducibility.

CAPLEX is not the first effort to standardize the naming and conventions for contrast agent-based perfusion MRI. In 1999, Tofts et al. introduced conventions for DCE-MRI, focusing on Tofts kinetic modeling.^19^ In this manuscript, we vastly extend and update this work by defining for the first time standard terminology for DSC-MRI, defining standards for additional perfusion-related quantities and models not included within the original 1999 Tofts et al. manuscript, and defining processes and models such that entire DCE/DSC analysis pipelines can be described in standardized terminology, not just the quantities involved. The structure of the lexicon has been designed such that it is easily extendable, and it is our plan to continually update and adapt its content in response to community input, and to ensure it is commensurate with state-of-the-art as the field progresses.

Furthermore, we also provide guidelines for how to use the lexicon to report DCE/DSC methods and results through two new reporting formats: lexicon-linked free text and the lexicon-linked XML pipeline format. Lexicon-linked free-text is designed to improve upon existing free-text descriptions commonly found in manuscripts or software documentation. It requires the user to report analyses and results using CAPLEX-defined names; link quantities, processes, and models to their lexicon definition using hyperlinks; and to include units for all quantities. The lexicon-linked XML pipeline format provides a means to describe analysis pipelines in standardized pseudo-code. The user begins by selecting the required high-level process (e.g., EstimateT1), then the lexicon definitions and XML schema guide the user through selection of additional processes, quantities, and models. The XML schema file defines rules to prevent illegal syntax or ordering of elements. Thus, it is much more difficult to omit vital information required by others to reproduce the work.

We have provided lexicon entries for quantities, models, and processes that are considered to be of primary importance to the DCE/DSC MRI community, with the minimum requirement that an end-to-end perfusion analysis pipeline can be encoded. The lexicon, however, is intended to be a dynamically growing inventory and has been designed such that it can be easily extended by users of the perfusion MRI community. We actively encourage members of the perfusion community to engage with the lexicon development by joining OSIPI Taskforce 4.2 or directly contributing to the GITHUB repository.

We provide terminology and guidelines for reporting but do not intend to give any recommendations on how to perform the analysis itself. It is the aim, however, to align the lexicon with initiatives providing such recommendations, and making sure the recommended analysis pipelines can be described using the lexicon. For example, we aim to ensure that the lexicon items cover the analysis processes recommended by the QIBA initiative for DCE and DSC workflows.^13,14^ It is also our plan to extend lexicon quantities and processes, ensuring compatibility with existing open-source processing pipelines as collated by OSIPI Taskforce 1.2 and new tools developed by OSIPI Taskforce 2.3.^22^ Furthermore, we hope that our process definitions and XML pipeline format may provide manufacturers of MRI scanners a means to document the analysis steps of their proprietary software in a way that enhances transparency for the end-user but does not compromise intellectual property. The lexicon could also be used to improve data sharing. OSIPI Taskforce 4.2 is currently in the process of amending the DICOM standard for the communication of DICOM parametric maps.^23^ With a standard description of perfusion-related metadata using lexicon terms, this will facilitate data sharing of derived parametric maps in a way that ensures the output data are linked to the analysis pipeline that was used to generate it. All these potential applications will be explored in the next 2-year cycle of the OSIPI (2023-2025).

## Figures and Tables

**TABLE 1:** List of compartment subscripts for indexing quantities defined in Section Q

Compartment code	Definition	Description
t	Tissue of interest	The tissue of interest
c	Capillary	The component of the vascular tree that lies between arterial supply and venous drainage
a	Feeding artery	The vessel feeding indicator to the tissue of interest
v	Draining vein	A vessel draining indicator from the tissue of interest
p	Blood plasma	The extracellular component of blood
b	Whole blood	The combined cellular and plasma components of blood
i	Intracellular space	The space that is contained within cell membranes
e	Extravascular extracellular space	The extracellular space within tissues excluding blood; also commonly referred to as the interstitial space
No index given	General case describing any or all voxels in an image	In some cases, it is useful to describe processes applied to any or all voxels in an image, regardless of location (e.g., computing a $T_{1}$ map); in this instance, no index should be given

**TABLE 2:** Lexicon entry for the blood plasma flow quantity

Code	OSIPI name	Alternative names	Notation	Description	OSIPI units	Reference
Q.PH1.002	Blood plasma flow	–	$F_{p}$	Volume of blood plasma flowing into a unit tissue volume per unit time; the flow inputs and exits the capillary vasculature	mL/min/100 mL	20

**TABLE 3:** Lexicon entry for the Tofts model process

Code	OSIPI name	Alternative names	Notation	Description	Reference
M.IC1.011	Tofts model	Kety model Generalized kinetic model	TM	The Tofts model describes bi-directional exchange of indicator between vascular and extravascular extracellular spaces (EES). The capillary compartment is assumed to have negligible volume. The EES is modeled as a well-mixed compartment. This forward model is given by the following differential equation: $\frac{dC_{t}}{dt}(t)=K^{\text{trans}}C_{a,p}(t)-(K^{\text{trans}}∕v_{e})\;C_{t}(t)$	21
				The impulse response function is given by: $I(t)=K^{trans}e^{\text{-}\frac{K^{trans}}{v_{e}}}t$ with $\left[C_{a,p}(t)\;(\text{Q.IC}1.001.[a,p]),\;\;t\;\;(\text{Q.GE}1.004)\right]$, $\left[C_{t}\;\;(\text{Q.IC}1.001.[t]),t\;(\text{Q.GE}1.004)\right]$, $\left[I\;(\text{Q.IC}1.005),\;t\;(\text{Q.GE}1.004)],K^{trans}(\text{Q.PH}1.008\right)$ and $v_{e}\;(\text{Q.PH}1.001.[e])$.	

## Data Availability

The current version of the ISMRM OSIPI contrast agent-based perfusion lexicon (CAPLEX) can be found here: https://osipi.github.io/OSIPI_CAPLEX.
